# A Digital Workflow for Virtual Articulator Mounting Using Face Scan and Facebow Capture: A Proof-of-Concept

**DOI:** 10.3390/dj13080378

**Published:** 2025-08-20

**Authors:** Giuseppe D’Albis, Marta Forte, Laura Stef, Diana Ramona Feier, Victor Diaz-Flores García, Massimo Corsalini, Saverio Capodiferro

**Affiliations:** 1 Department of Interdisciplinary Medicine, University of Bari Aldo Moro, 70121 Bari, Italy; marta.forte@uniba.it (M.F.); massimo.corsalini@uniba.it (M.C.); saverio.capodiferro@uniba.it (S.C.); 2 Faculty of Medicine, Lucian Blaga University Sibiu, 550169 Sibiu, Romania; laura.stef@ulbsibiu.ro; 3 Faculty of Medicine, Dimitrie Cantemir University, 540545 Târgu Mureş, Romania; dr.ramonafeier@yahoo.ro; 4 Department of Pre-Clinical Dentistry, School of Biomedical Sciences, Universidad Europea de Madrid, 28670 Villaviciosa de Odón, Spain; victor.diaz-flores@universidadeuropea.es

**Keywords:** virtual articulator, facebow, digital dentistry, CAD-CAM, facial scan

## Abstract

**Objectives:** This article introduces a digital technique for virtual articulator mounting by employing the scan of a facebow worn by the patient as a virtual reference. **Methods:** The digital technique enables the transfer of the maxillary arch orientation relative to the cranial base into a CAD-CAM environment (Ceramill Mind; AmannGirrbach), without the need for ionizing radiation or identification of facial landmarks. By digitally aligning the intraoral scans of the dental arches (Trios 4; 3Shape) with a 3D facial scan and the scanned facebow in position (Artex; AmannGirrbach), clinicians can reproduce the cranium-to-maxilla spatial relationship accurately and intuitively. **Results:** This radiation-free protocol provides virtual cross-mounting and allows for the use of a semi-adjustable articulator within common CAD-CAM software. **Conclusions:** Given that intraoral scanners, facial scanners, and design software with articulator simulation are becoming more available in modern clinical workflows, this method introduced here could be a viable radiation-free and easy-to-use alternative. However, larger cohorts and standardized testing protocols are needed to determine its clinical reproducibility and reliability.

## 1. Introduction

Accurately positioning the maxillary and mandibular arches on the articulator in accordance with the patient’s anatomical reference planes and mandibular hinge axis is a fundamental step in the diagnosis and prosthetic management of complex rehabilitations [1,2,3].

The evolution of digital technologies and the development of CAD-CAM systems have introduced a valid virtual alternative to conventional mechanical articulators [4,5]: the virtual articulator [6,7]. This tool enables a digital simulation of mandibular movements and occlusal contacts within a virtual environment, while preserving the spatial relationship between the jaws [8,9,10].

In conventional workflows, the use of a facebow is essential for transferring the spatial orientation of the maxillary arch relative to the cranial base. In the digital context, the challenge lies in replicating this orientation virtually by referencing anatomical planes from the patient’s head. Among these, CBCT-based alignment protocols are recognized for their high accuracy in transferring digitized models to the virtual articulator, especially in orthognathic surgery and interdisciplinary cases [11,12,13]. Alternative methods rely on extraoral anatomical references and facial scans, employing tools such as 3D surface scanners, axiographic systems, photogrammetry, digital photography, and stereophotogrammetry [14,15,16,17,18,19].

The use of 3D imaging, also in orthodontics, has significantly enhanced diagnostic precision and treatment planning by enabling the simultaneous capture of both facial structures and dentition. Unlike traditional 2D photographs and radiographs, 3D imaging offers a comprehensive volumetric representation of the patient’s craniofacial anatomy, allowing clinicians to evaluate skeletal symmetry, soft tissue contours, and dental alignment with greater accuracy. Technologies such as facial scanners and intraoral scanners can be integrated to produce a unified digital model, facilitating in-depth analyses of occlusion, airway space, and esthetic relationships. This holistic, data-rich approach supports individualized, patient-centered treatment planning and strengthens communication between the orthodontist, the patient, and the interdisciplinary team [20,21].

The integration of digital protocols into prosthodontic and maxillofacial procedures has revolutionized clinical procedures by enhancing accuracy, reproducibility, and efficiency. Mechanical articulators, while sufficient, are prone to operator variability and require tedious analogous steps. Digital articulators, however, offer the potential for standardized procedures for jaw relation transfer, dynamic occlusal analysis, simulation of functional movement, and improvement of clinician–laboratory communications. The recent literature points to the requirement for precise virtual articulation as a prerequisite to expected outcomes of full-arch rehabilitations, implant planning, and splint therapy. However, the absence of a digitally equivalent facebow with universal consensus has restricted global application, so ongoing research investigates other acquisition alternatives bridging the analog-digital workflow gap.

The authors introduce a digital, radiation-free proof-of-concept approach to virtual articulator mounting. In this study, the null hypothesis assumes that the proposed digital technique, which is based on the integration of facial scanning and scanned facebow data, does not enable accurate orientation of digital dental casts within a virtual articulator without the use of radiographic imaging. In contrast, the alternative hypothesis proposes that this fully digital protocol provides a reliable method for aligning the maxillary arch in relation to the cranial base using non-invasive digital data alone. This workflow represents an innovative alternative both to radiation-based techniques and to traditional analog methods, offering a simplified and accurate solution for virtual articulation.

## 2. Materials and Methods

The technique was clinically implemented by a dental practitioner (G.D.) to perform a fixed prosthetic rehabilitation. The methodology was applied to a single patient. The clinical procedure was explained to the patient, who provided written informed consent, including consent for the publication of their facial image. This digital protocol was carried out using commonly available equipment in modern dental practices, including an intraoral scanner (TRIOS; 3Shape, Copenhagen, Denmark), a facial scanning device (iPad Pro 11; Apple Inc., Cupertino, CA, USA), and CAD-CAM software (Ceramill mind; AmannGirrbach, Koblach, Austria) with a virtual articulator module.

### 2.1. Chairside Clinical Procedure

Maxillary and mandibular arches were scanned using an optical intraoral scanner (Trios 4; 3Shape) to capture their morphology and register their occlusal relationship. The scans were exported in Polygon File Format (PLY) to preserve color and texture data. Subsequently, an extended scan was performed to capture the perioral soft tissues, the nasal region, and the incisal third of the face, allowing for later alignment with extraoral facial scans [22]. A full-face scan was then acquired with the patient smiling, using a dedicated 3D scanning application (Bellus3D FaceApp 2.0.3; Bellus3D Inc., Cupertino, CA, USA) on a tablet device [23].

The resulting mesh file was exported and optionally refined using 3D mesh editing software (MeshMixer^®^ 3.5.474; Autodesk, San Rafael, CA, USA) to isolate the facial scan by removing the dental arches and defining the labial boundaries. Then, an Artex facebow (AmannGirrbach, Koblach, Austria) was assembled and positioned on the patient, ensuring stability at the external auditory meatus and glabella [24]. A second facial scan was then performed while the facebow was in place, enabling digital capture of its position in relation to the patient’s head. A matting spray could be applied to prevent scanning distortions, considering that the facebow material is metallic.

### 2.2. Digital Workflow in CAD/CAM Software

All scans were imported into the CAD-CAM software (Ceramill mind 4.8; AmannGirrbach, Koblach, Austria) for alignment. The following sequence of superimpositions was carried out:

- Alignment of the intraoral scan to the perioral scan using the teeth as reference landmarks to ensure accurate superimposition.
- Superimposition of the perioral scan onto the full-face scan by referencing shared anatomical landmarks on the nose, eyebrows, and lips, allowing for precise integration of the two datasets.
- Registration of the facebow-in-place scan to the facial scan dataset using common anatomical landmarks to ensure precise alignment.

The virtual articulator (Artex CR; AmannGirrbach, Koblach, Austria) was then aligned within the software based on two main references:

- The hinge axis, corresponding to the tips of the ear rods on the facebow, was matched with the articulator’s rotation axis.
- The horizontal reference plane of the facebow was oriented parallel to the base of the articulator.

**Figure 1:**
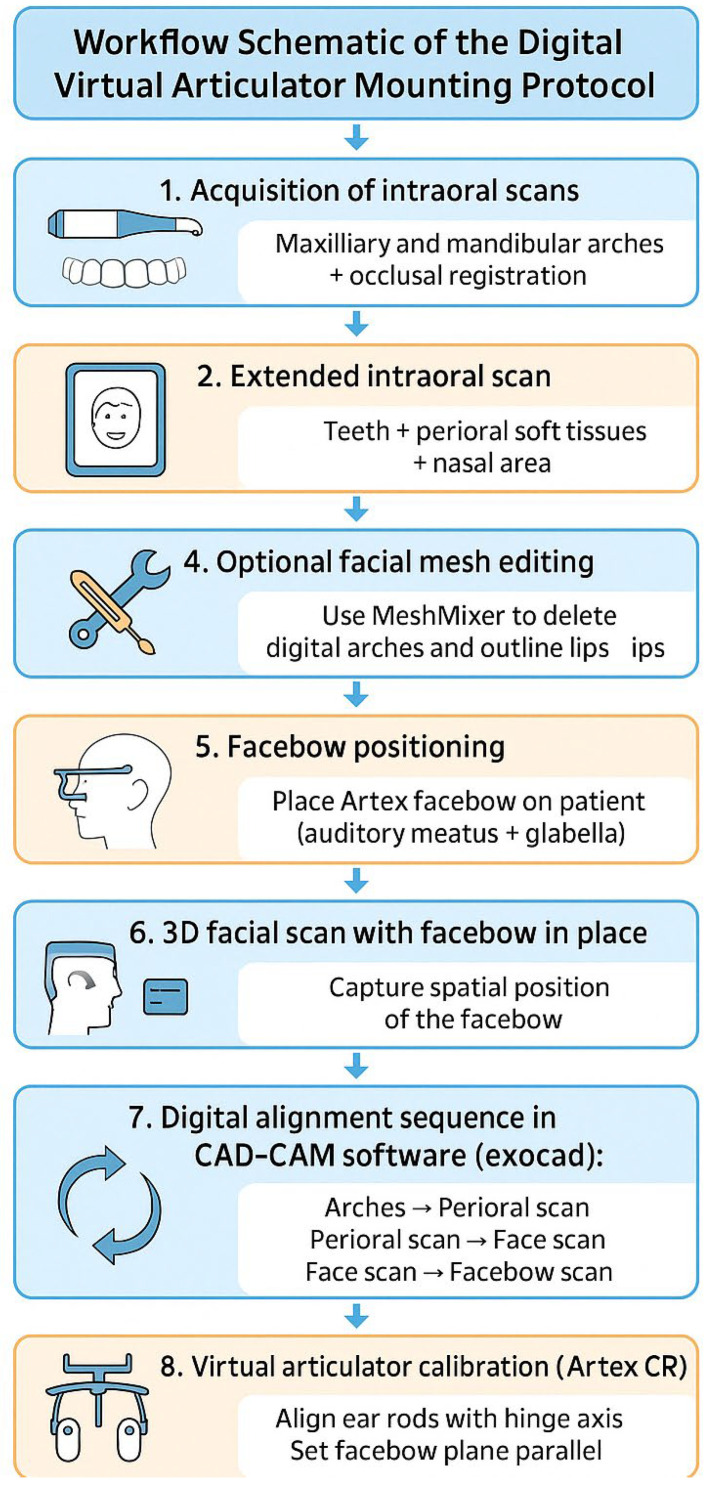
Infographic-style diagram of the digital workflow.

This alignment enabled the digital cross-mounting of all records and the virtual simulation of mandibular dynamics using a semi-adjustable articulator in a completely digital workflow. The entire process is summarized in the following graphic representation (Figure 1).

### 2.3. Accuracy and Repeatability of the Protocol

The spatial position of the maxillary arch was recorded using an analog facebow (Artex Facebow, AmannGirrbach) according to the manufacturer’s protocol. Specifically, after obtaining cusp–tip impressions of the upper arch in wax on the bite fork, the facebow was positioned on the patient with the earpiece locators in the external auditory canals and a stabilizing nasion support. The bite fork was then attached to the facebow via the three-dimensional universal joint and locked in position. The facebow record obtained was transferred to a semi-adjustable articulator (Artex CR, AmannGirrbach) using the dedicated transfer jig with a magnetic base. This allowed the plaster cast of the maxilla to be mounted on the physical articulator, faithfully reproducing the spatial orientation captured on the patient.

Subsequently, the mounted plaster models of the maxillary and mandibular arches were digitized using a bench-top optical scanner (Ceramill mind, AmannGirrbach). This produced 3D digital models that were imported into a virtual articulator software configured with parameters corresponding to the Artex CR articulator. In this way, the alignment and movement settings of the Artex CR were preserved in the virtual environment, maintaining the proper occlusal relationships.

To verify the correctness of alignments and the fidelity of the maxillary position in the virtual articulator relative to the physical reference, the transfer procedure to the digital domain was repeated four times for the same patient. Using the same intraoral and extraoral scans, virtual articulator mounting was performed four times by four different clinicians, all experienced in CAD-CAM technologies (G.D., M.F., M.C., S.C.). The repetition of the protocol on the same subject allows for the assessment of the intrinsic variability and repeatability of the digital workflow, in accordance with previously published methodologies for evaluating the precision of digital facebow systems.

To quantify the differences between the reference analog mounting and the four repeated virtual mountings, five anatomical landmark points on the maxillary arch were identified:

- RM: mesiobuccal cusp of the right first molar
- RC: cusp tip of the right canine
- CI: labial surface of the right central incisor
- LC: cusp tip of the left canine
- LM: mesiobuccal cusp of the left first molar

For each of these points, the linear distance (in millimeters) was measured between its location on the analogic-mounted maxillary cast and its corresponding location in each of the four virtual mountings. This analysis enabled evaluation of the deviation of the maxilla’s position in the virtual articulator relative to the physical reference, providing a measure of the mounting accuracy trueness and the repeatability in the virtual environment. The trueness was defined as the deviation of the maxillary position in the virtual records compared to the analogic mounted cast reference, and the precision was defined as the variation among the repeated virtual mounting. Each linear deviation for the defined points was thus calculated to assess how closely the virtual mountings replicated the reference facebow mounting and how consistent the virtual mounting procedure was across multiple trials.

## 3. Results

**Figure 2:**
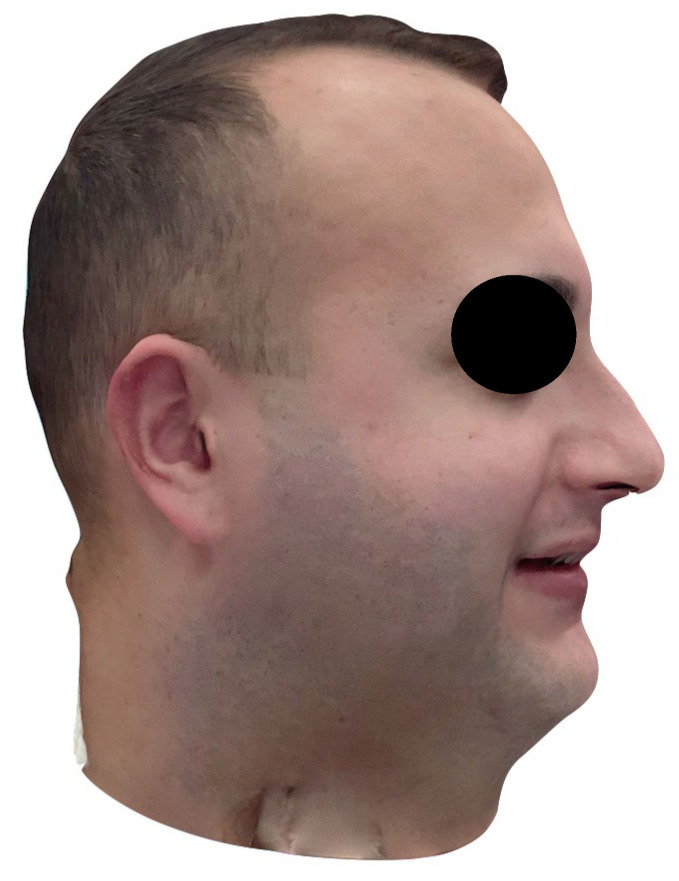
Facial scan acquired using an iOS device.

**Figure 3:**
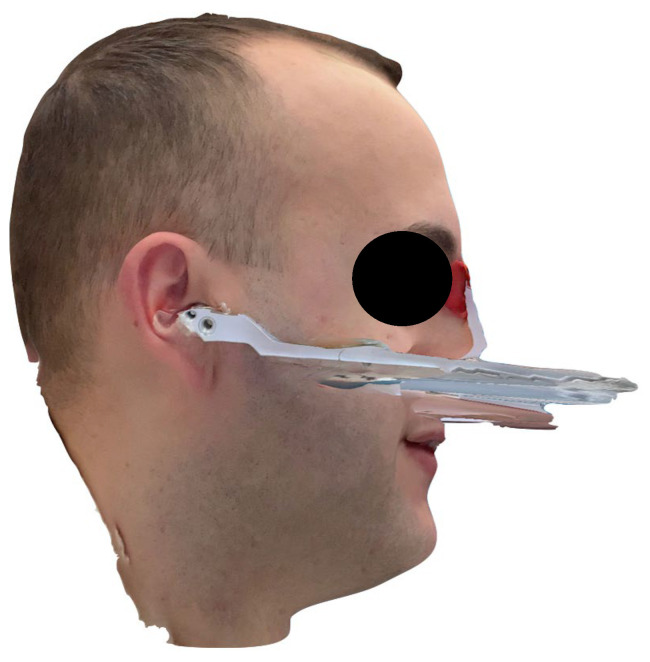
Facial scan performed with the facebow properly worn according to the manufacturer’s guidelines.

The fully digital facebow workflow proved feasible and user-friendly in a clinical setting. The protocol was straightforward to implement, integrating smoothly with existing intraoral scanning and CAD/CAM tools. Notably, the process did not require any cone-beam CT or extra radiographs, which improved patient comfort and eliminated radiation exposure. The 3D facial scan of the patient, as well as the scan with the facebow in place, were each completed in approximately 60 s, highlighting the user-friendly nature of facial scanning applications for clinical use (Figure 2 and Figure 3). However, minor technique sensitivities were noted; for instance, careful attention was required to avoid tracking errors when scanning the facebow (due to its reflective metal surface) and to ensure the face remained immobile during the facial scan.

**Figure 4:**
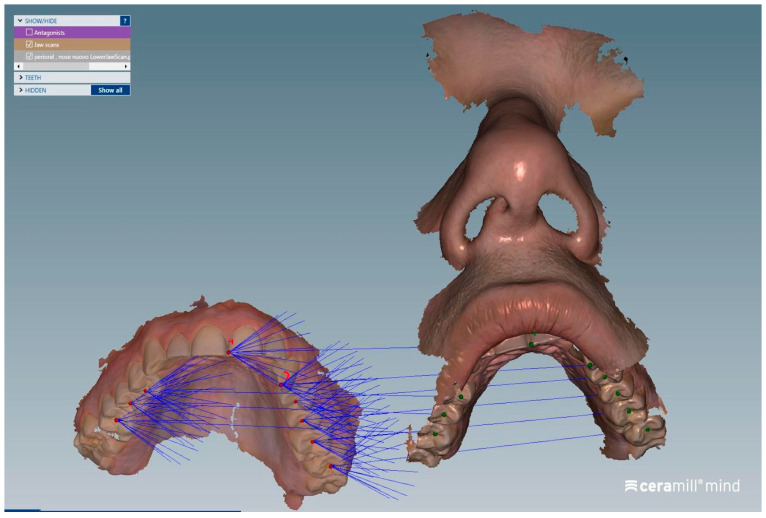
The alignment workflow of the upper arch scan with the perioral soft tissue scan was performed using the teeth as reference landmarks to ensure accurate superimposition.

**Figure 5:**
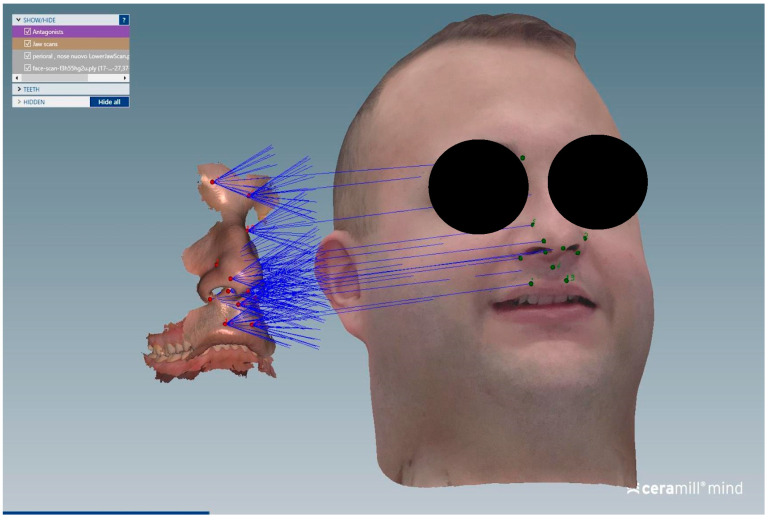
The alignment between the perioral soft tissue scan and the full facial scan was performed by referencing shared anatomical landmarks on the nose, eyebrows, and lips, allowing for precise integration of the two datasets.

**Figure 6:**
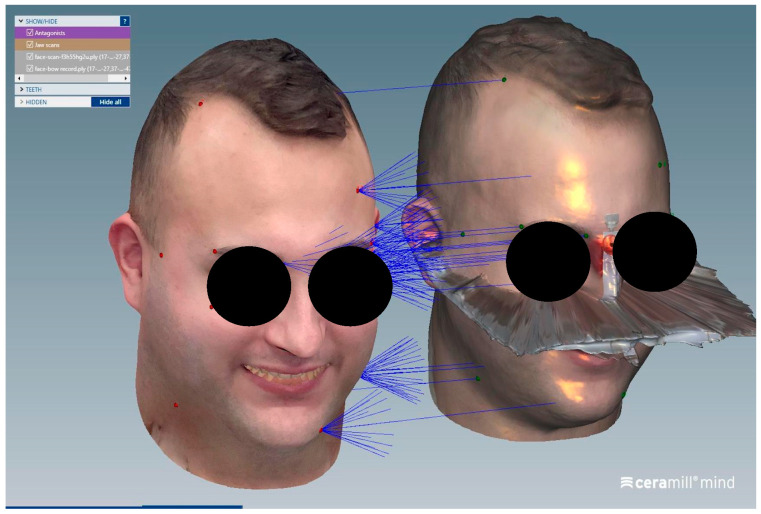
Superimposition of the face-only scan with the facial scan acquired while the facebow was in place was performed using common anatomical landmarks to ensure precise alignment.

The matching steps of all scans, namely the alignment of the upper arch scan with the perioral soft tissue scan (Figure 4), the alignment between the perioral soft tissue scan and the full facial scan (Figure 5), and the superimposition of the face-only scan with the facial scan acquired with the facebow in place (Figure 6), were performed smoothly using the CAD/CAM software. Common anatomical landmarks were manually selected, and the ‘best match’ function was employed to facilitate the process. This phase required approximately 2 min to complete.

**Figure 7:**
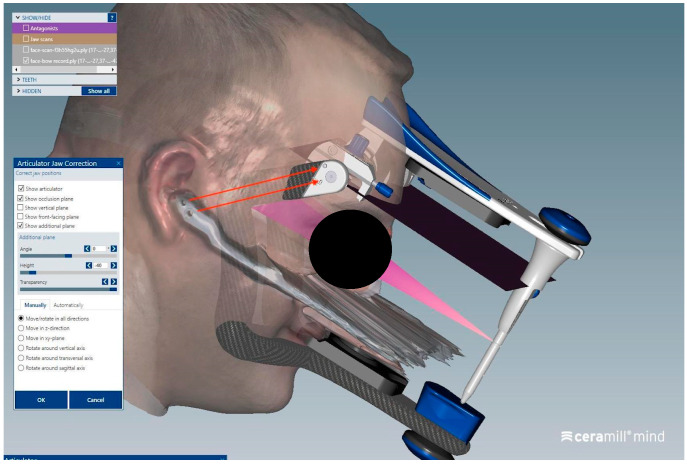
This image illustrates the movement needed to align the facial scan with the digital facebow by matching the two markers to their counterparts on the virtual articulator.

**Figure 8:**
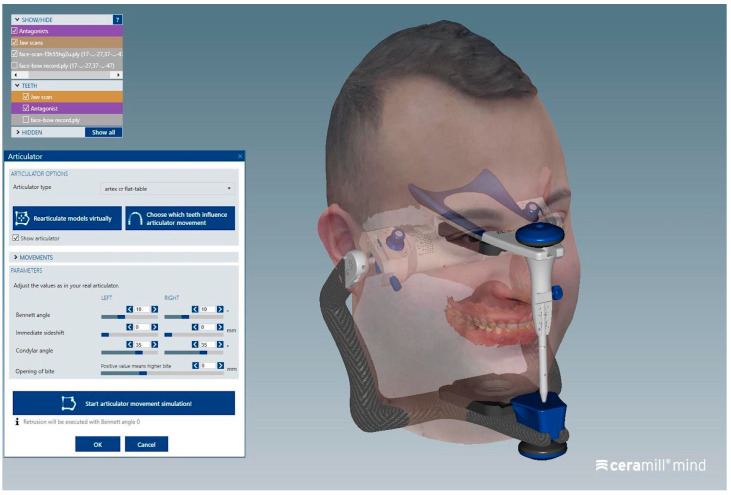
Finalized virtual articulator mounting with complete alignment of all reference data.

In contrast, the orientation phase of the dental arches within the virtual articulator, specifically the alignment of the two facebow markers with the corresponding markers on the digital articulator, can be considered operator-dependent, as it involved manual positioning of the scans (Figure 7 and Figure 8).

The digital protocol offered practical advantages: all records were in STL/OBJ format, making them easy to share with the dental laboratory and to archive for future reference, unlike analog facebow transfers which require physical storage of facebow indices.

When compared to conventional analog facebow mounting, the digital facebow protocol appears promising in transferring the maxillary cast orientation; however, further studies are needed to comprehensively evaluate its accuracy. In our cases, the virtually mounted maxillary arch, aligned using the facial scan and digital facebow reference, closely matched the patient’s anatomical orientation. No significant discrepancies were observed when compared to an analog facebow mounting. Traditional facebow transfer requires placing a physical facebow on the patient to record the hinge axis and an anterior reference point, then mounting plaster casts on a mechanical articulator. Clinicians noted that this process can be time-consuming and sometimes uncomfortable for the patient, especially if repeat impressions or remounting are needed. The digital workflow, by avoiding plaster casts and physical facebows beyond a brief scanning step, reduced chair-time and patient inconvenience. Additionally, the digital method allows immediate verification of the cast orientation: once the scans are aligned, the position of the virtual cast relative to cranial landmarks can be visualized and adjusted in software in real time. This is an improvement over the analog method, where errors in mounting often go undetected until the prosthetic fabrication stage.

Some digital workflows have used CBCT imaging of the patient wearing a radiographic index or facebow to locate the maxillary cast in 3D. While geometrically effective, CBCT methods have downsides: they expose the patient to radiation and require segmentation of the dentition from the scan, and they demand access to expensive imaging hardware. In our protocol, no CBCT was needed; all reference data came from optical scans, thus avoiding unnecessary radiation. Likewise, jaw-tracking devices (digital axiographs) can record patient-specific hinge axes and movements, but these systems (e.g., optical jaw trackers) tend to be specialized and costly, and their accuracy for cast mounting is not yet well documented. By contrast, the facebow-guided facial scanning method relies on equipment already common in many digital dentistry practices (intraoral and face scanners), making it a cost-effective solution.

### Results of Accuracy and Repeatability of the Protocol

**Figure 9:**
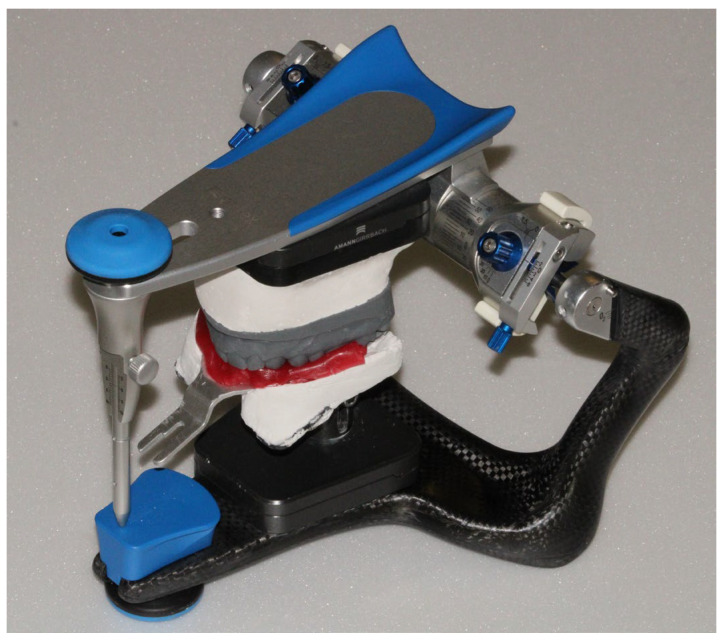
Mounting of the upper arch on the physical articulator.

**Figure 10:**
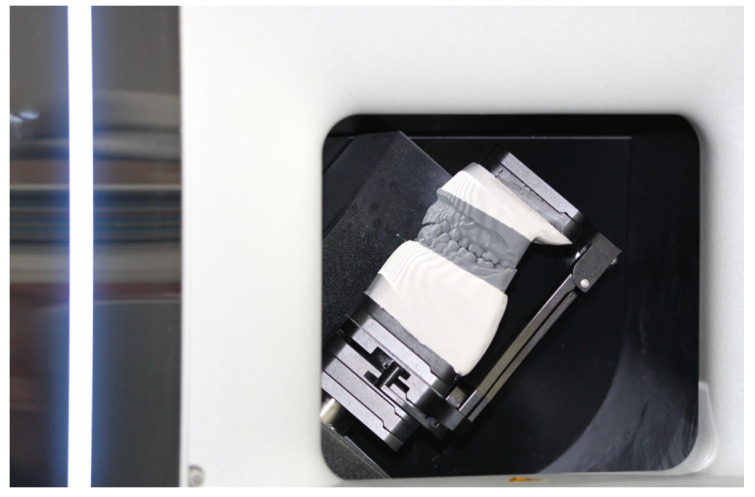
Scanning of the models with the analog-recorded orientation and in maximum intercuspation.

The position of the maxilla was recorded using a facebow, and the models in maximum intercuspation were mounted on an Artex CR articulator according to the manufacturer’s instructions (Figure 9). The models were scanned using a desktop scanner (Figure 10). Thus, the orientation of the upper arch was obtained through the analog workflow (Model 0).

The virtual articulator mounting using facial scanning with the facebow in place was repeated four times by four different clinicians. The average time for manual orientation of the scan was 35 s.

The following results report the measurements taken at the same anatomical landmarks, comparing the digitally scanned cast mounted using the analog protocol with each of the four models mounted using the digital protocol by four different clinicians (Table 1).

**Figure 11:**
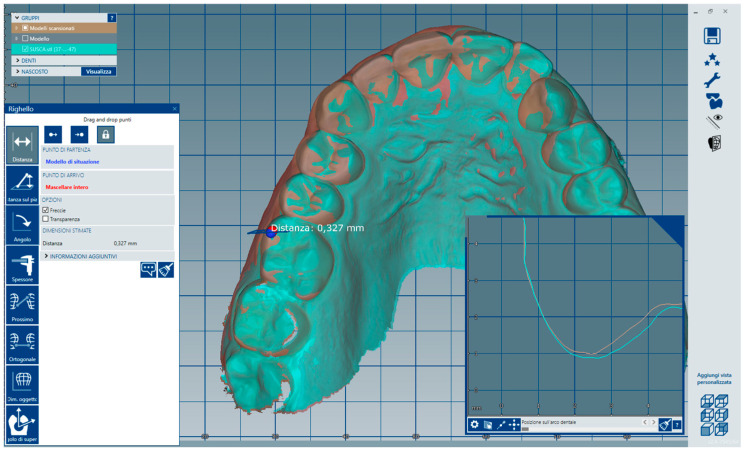
The image shows the distance in millimeters between Model 0 and Model 1 at point RM, measured in the software using the ‘ruler’ tool.

**Figure 12:**
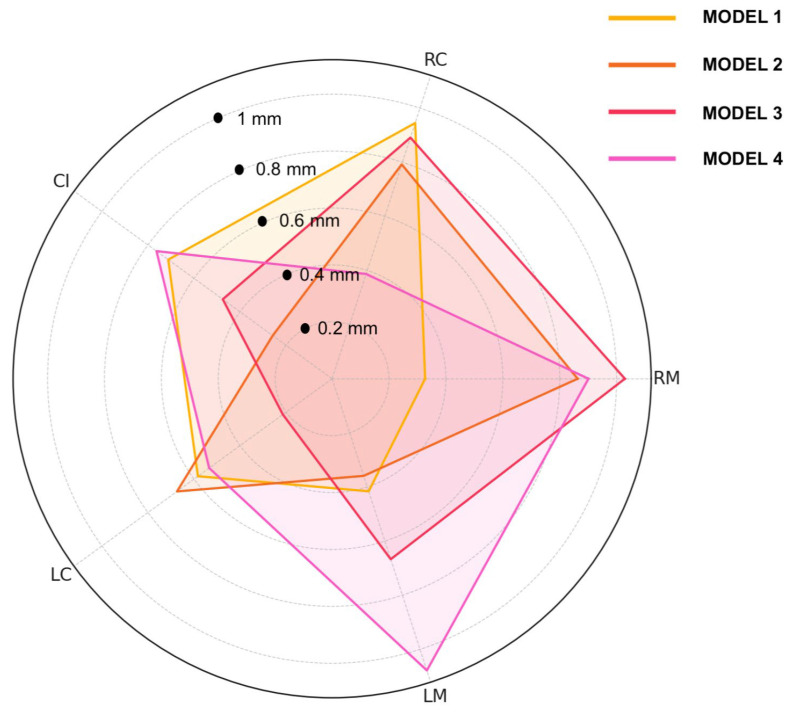
Radar plot representing the distances (in mm) from the analog-mounted model for each reference point (RM, RC, CI, LC, LM) across the four digitally mounted models.

The radar plot below displays the deviations (in mm) for five anatomical reference points (RM, RC, CI, LC, LM) comparing the analog-mounted model to four digitally mounted models (Figure 11). Model 3 exhibited the highest deviation at the RM point (1.029 mm), while the lowest deviation across all models was observed at the LC point in Model 3 (0.214 mm). RC deviations ranged from 0.387 mm (Model 4) to 0.945 mm (Model 1), and CI values varied between 0.258 mm (Model 2) and 0.763 mm (Model 4). LC deviations were consistent, ranging from 0.214 mm to 0.674 mm. For LM, the smallest deviation was 0.359 mm (Model 2), and the largest was 1.078 mm (Model 4). These results demonstrate that despite slight inter-operator variability, the deviations across all points and models remained within approximately 1 mm, supporting the reliability and reproducibility of the digital facebow protocol (Figure 12).

The linear deviations measured at each anatomical landmark between the virtual mountings and the analog-mounted reference model were consistently within the sub-millimeter range. The maximum discrepancy observed was approximately 1.78 mm, while most points showed differences of only a few tenths of a millimeter. These small deviations indicate a high level of trueness, suggesting that the digital mounting technique can closely replicate the spatial orientation achieved with the conventional facebow.

Similarly, the variation among the four repeated virtual mountings was minimal. Differences across trials remained within a few tenths of a millimeter, demonstrating excellent precision and repeatability of the virtual workflow. This low intra-procedural variability confirms that the digital technique produces consistent maxillary positioning across multiple executions. Overall, the results for trueness and precision suggest that the proposed virtual mounting protocol can accurately and reliably reproduce the maxillary position recorded with the analog facebow, offering a valid and repeatable alternative for digital articulation (Table 2).

## 4. Discussion

The present article introduces a fully digital, radiation-free workflow for transferring the spatial orientation of the maxillary arch into a semi-adjustable virtual articulator. By capturing the arbitrary axis directly through the digitalization of a facebow worn by the patient, this technique eliminates the need for ionizing imaging modalities such as cone-beam computed tomography (CBCT), thus significantly reducing patient exposure to radiographic procedures.

One of the main advantages of this approach lies in its non-invasive and device-free workflow for matching intraoral scans with the patient’s facial scan. Unlike other protocols that rely on external radiographic markers or fiducial devices, the method described herein utilizes readily available digital data, intraoral scans, 3D facial scans, and the extraoral scan of the facebow in position, making the process safer and more intuitive for both clinician and patient. Furthermore, the integration of facial scans within the CAD-CAM planning software can enhance diagnostic value, clinical communication, and treatment visualization, particularly in interdisciplinary cases [25].

Despite these advantages, certain limitations must be acknowledged. First, the protocol requires the use of multiple digital platforms, including intraoral scanning software, 3D facial scanning applications, mesh editing tools, and advanced CAD software with virtual articulator modules. This multisystem integration may be technically demanding and time-consuming, particularly for clinicians unfamiliar with digital workflows. Additionally, the learning curve associated with 3D mesh alignment and virtual articulator calibration can be steep, necessitating adequate training and clinical experience to ensure reliable outcomes [26].

Another limitation is the dependence on facial scanning hardware and software, which may not yet be universally available in all clinical settings. Although devices (such as tablets) equipped with dedicated scanning applications are becoming increasingly accessible, their consistent accuracy and repeatability must be validated across different populations and facial morphologies [27].

The workflow described in this study was developed using a specific facial scanning software, which, at the time of writing, is not available or supported in certain countries due to regulatory or commercial limitations. This may represent a barrier to the widespread adoption of the proposed protocol in some clinical contexts. However, the increasing interest in facial scanning for dental applications has led to the development of several alternative solutions. These include both mobile applications capable of generating accurate 3D facial models and dedicated extraoral scanners designed specifically for dental and maxillofacial use. Many of these systems offer integration with CAD software and virtual articulators, making them suitable for use in prosthetically driven workflows. While software availability may vary geographically, the core principles of the workflow remain applicable and can be adapted to other platforms offering comparable accuracy and compatibility with digital prosthetic planning tools.

Moreover, several factors may affect the accuracy of facial scan integration with a facebow. One common source of error is the reflectivity of the facebow surface, which can interfere with scan data acquisition by generating artifacts or incomplete geometry. This can be mitigated by using facebows with matte finishes or applying a scanning spray to reduce reflections and improve surface detection.

Patient movement during scanning is another significant variable, especially during the alignment of multiple datasets. To minimize motion artifacts, it is essential to provide clear instructions to the patient, reduce scan duration, and, when possible, use head stabilization techniques. Proper pre-scan calibration, optimal lighting conditions, and adherence to standardized scanning protocols further help reduce variability and improve reproducibility in clinical applications.

This digital method is especially indicated in cases where there is a need to reproduce the patient’s functional movements within a digital environment using average anatomical values, rather than patient-specific motion capture. As such, it is suitable for a wide range of clinical applications, including fixed prosthodontics, implant-supported prostheses, gnathological assessments, and orthodontic planning [28].

In conclusion, while the described protocol offers clear benefits in terms of patient safety, workflow simplification, and digital integration, its implementation should be considered within the context of each clinic’s technological resources and the practitioner’s digital competence. Further studies are warranted to validate the reproducibility of this technique and explore its full potential in routine clinical practice. As digital dentistry continues to evolve, the integration of facial scanning, intraoral data, and virtual articulators will become a vital component of comprehensive treatment planning. The protocol outlined in this manuscript represents a step toward a more unified digital workflow, where patient-specific anatomical references can be incorporated without the need for radiographic exposure or complex physical devices [29].

A potential future development of interoperable software platforms can further streamline the process, reducing the need for third-party mesh editing software and enabling real-time integration of intraoral and facial scans within a single interface. Additionally, artificial intelligence and machine learning algorithms can also automate the scan alignment, anatomical landmark identification, and virtual articulator calibration from predictive models, decreasing the operator-dependent variability and flattening the clinician learning curve [30,31]. Artificial intelligence applications are increasingly being integrated into facial scan alignment and virtual articulation workflows, enhancing accuracy and reducing operator dependency. By automatically identifying anatomical landmarks and optimizing superimposition, artificial intelligence-driven tools streamline the digital workflow and improve reproducibility [32].

The ongoing miniaturization and enhancement of 3D facial scanning technologies, such as smartphone-based or wearable scanners, can also broaden the availability of this method, rendering it practical in both general and specialized dental practices. As these devices are refined to be more precise, cost-effective, and easy to use, they will become instrumental in broadening the use of virtual articulator protocols in daily clinical practice. Moreover, research in the future must emphasize clinical verification of this method in various patient groups and treatment methods. Research establishing the reproducibility, precision, and clinical outcomes of virtual mounting procedures compared to conventional facebow records and registrations on the basis of CBCT will be essential for putting standardized protocols into practice [33].

Finally, future developments in motion capture technology, such as dynamic facial tracking and digital axiography, may ultimately enhance or possibly replace the current static procedures; the combination of facial motion data with intraoral/facial scans would open the door for completely individualized virtual articulators, which would be capable of replicating dynamic mandibular movement with great accuracy.

Recent literature has explored the accuracy of transferring maxillary casts to virtual articulators compared to conventional analog systems. Studies generally show that virtual articulators can reproduce jaw relationships with clinically acceptable accuracy. Reported deviations in position between analog and virtual setups vary depending on the workflow, software used, and type of reference markers.

In one clinical investigation, a hybrid workflow using scanned facebow-mounted casts demonstrated a mean deviation of 0.55 ± 0.31 mm, with 93% of contact points matching between analog and virtual articulators, and a maximum error of 1.02 mm, supporting its reliability in occlusal analysis [34]. Another in vitro study reported a mean discrepancy of 0.75 ± 0.46 mm when comparing conventional facebow transfer to a digital 3D scanning technique, suggesting that while acceptable for diagnostic use, such variance might not be ideal for high-precision procedures like orthognathic surgery [16].

Further validation was provided by studies evaluating occlusal records: one found a mean deviation of only 0.069 mm (SD 0.011 mm) between conventional and virtual intercuspal alignments, demonstrating high fidelity of virtual mounting in reproducing occlusal contacts [35]. Another study using transfer plates reported mean deviations of 0.08–0.09 mm in cast positioning, with excellent reproducibility (precision ~12 µm), and increased sensitivity in contact point detection after digital occlusal optimization [36].

The deviations observed in our study align with the range reported in previous digital methodologies, confirming that virtual mounting workflows can accurately replicate analog articulator positions. With deviations typically falling between 0.07 mm and 0.8 mm, these results reinforce the clinical reliability of such techniques when appropriate protocols are implemented.

## 5. Conclusions

While this approach currently offers advantages in terms of usability, safety, and digital integration, its full potential will be realized as advancements in software, scanning technologies, and artificial intelligence converge to enable seamless and patient-centered digital workflows.

The technique presented demonstrates a radiation-free method for virtual articulator mounting by utilizing facial scan data in combination with facebow positioning. Its integration within the digital workflow enhances clinical accuracy without requiring additional radiographic imaging or extraoral matching devices. Although certain limitations persist, such as software complexity and the need for operator expertise, the method represents a promising and accessible solution for the digital replication of mandibular dynamics in fixed prosthodontics, implantology, gnathology, and orthodontics. This preliminary proof-of-concept technique requires validation through larger patient cohorts and standardized evaluation protocols. Furthermore, comparative studies with existing methods, as well as further validation and technological refinement, are essential to assess its clinical relevance and potential advantages.

## 6. Patient

The clinical procedure was explained to the patient, who provided written informed consent, including consent for the publication of their facial image.

## Figures and Tables

**Table 1:** Distance in millimeters, for each reference point, from the digitally scanned model mounted using the analog protocol to the model mounted with the digital protocol.

	RM	RC	CI	LC	LM
Model 1	0.327 mm	0.945 mm	0.712 mm	0.583 mm	0.416 mm
Model 2	0.863 mm	0.792 mm	0.258 mm	0.674 mm	0.359 mm
Model 3	1.029 mm	0.891 mm	0.475 mm	0.214 mm	0.667 mm
Model 4	0.901 mm	0.387 mm	0.763 mm	0.534 mm	1.078 mm

**Table 2:** Mean and standard deviation of the measurements.

Landmark	Trueness (Mean Deviation, mm)	Precision (Std. Dev., mm)
RM	0.780	0.310
RC	0.754	0.233
CI	0.552	0.222
LC	0.501	0.197
LM	0.630	0.336

## Data Availability

The data presented in this study are available on request from the corresponding author due to the novelty of the technique and the use of different software.
